# Supplementary material for: Ergogenic Effect of BCAAs and L-Alanine Supplementation: Proof-of-Concept Study in a Murine Model of Physiological Exercise
Source: Nutrients. 2020 Jul 30;12(8):2295. doi: 10.3390/nu12082295 (PMC7468919; doi:10.3390/nu12082295)
Supplement: Supplementary file 1 [file nutrients-12-02295-s001.zip › nutrients-861838-supplementary.pptx]

## Slide 1
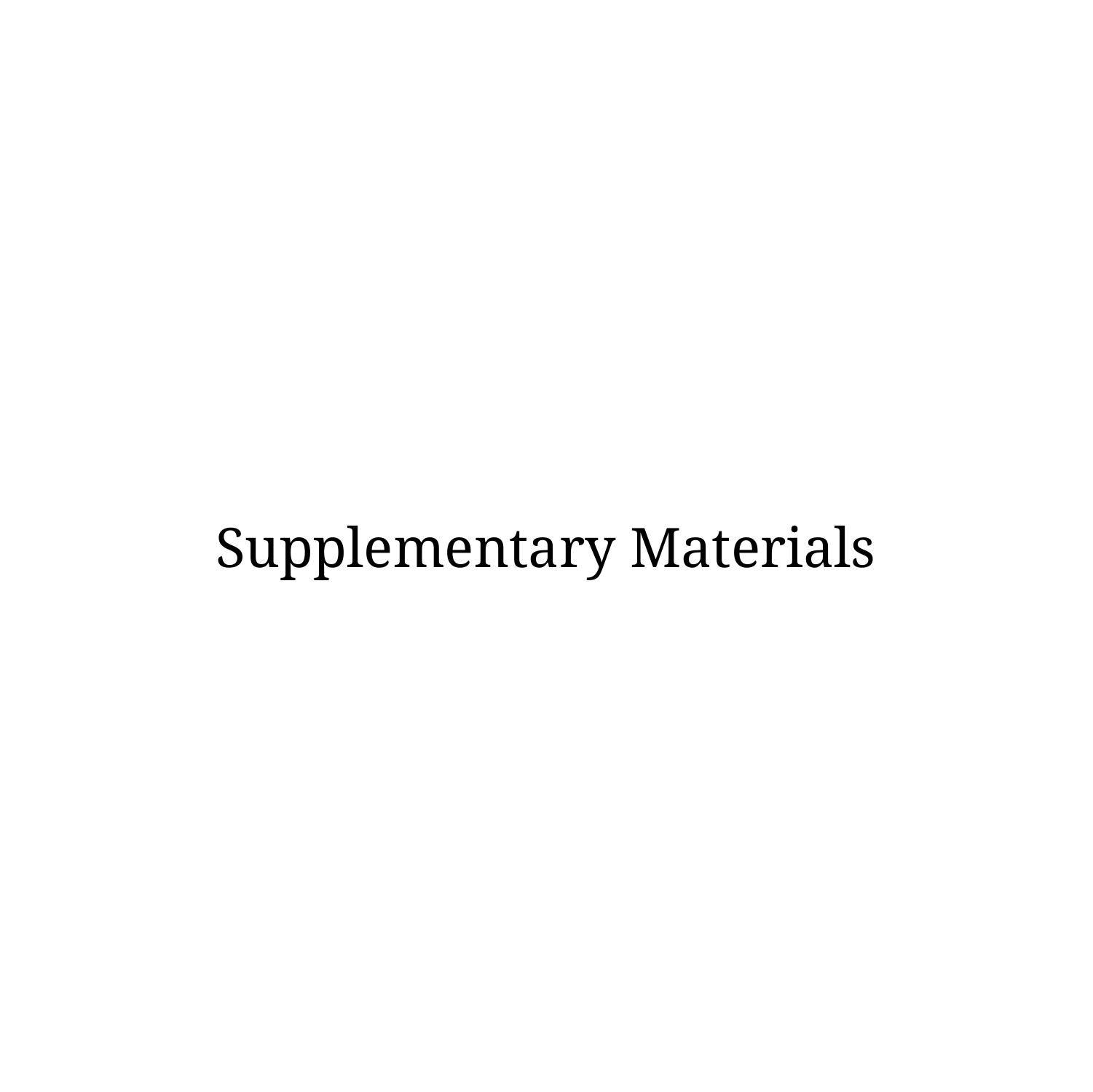

Supplementary Materials

## Slide 2
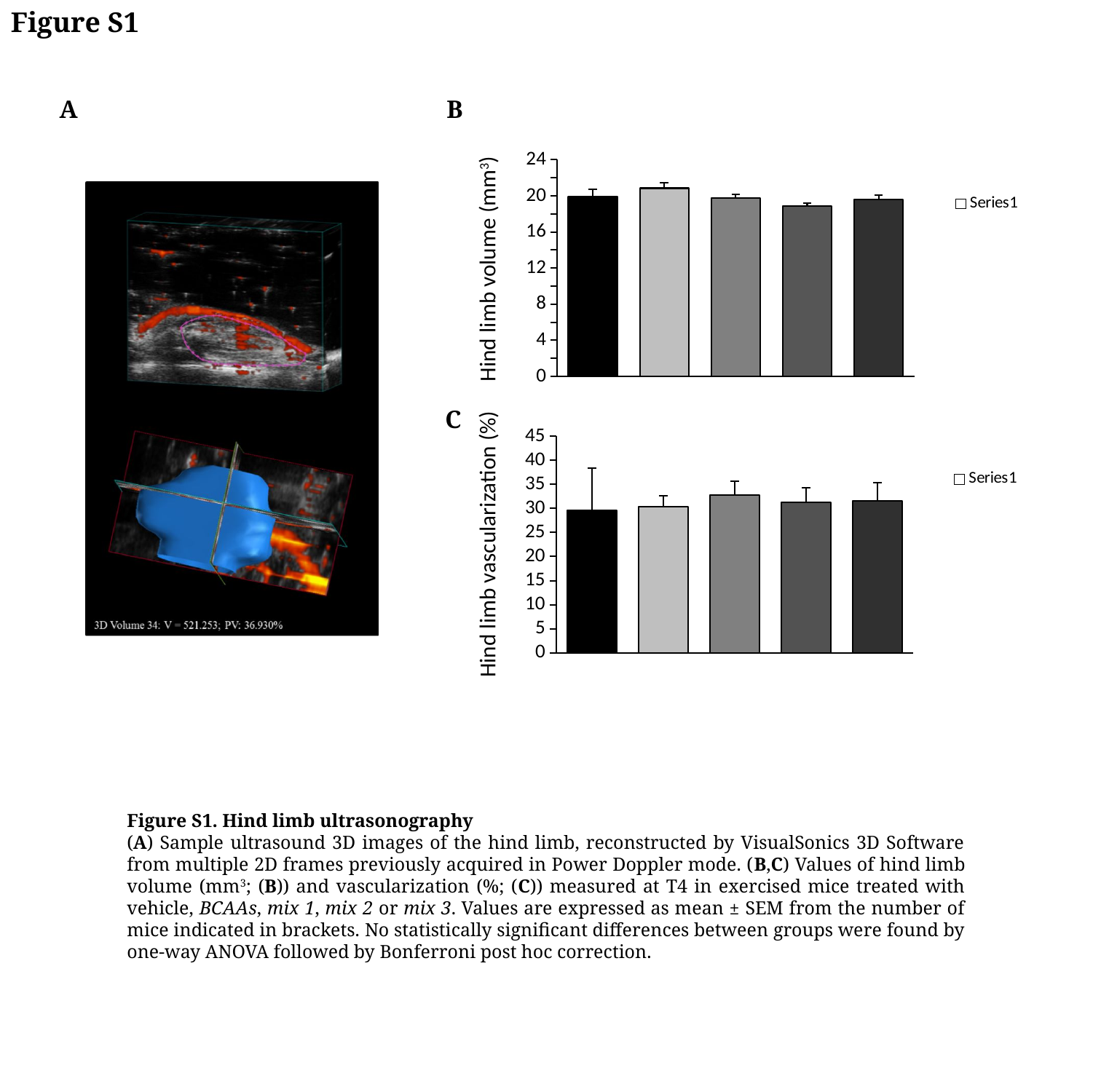

Figure S1
A
B
### Chart
| Category | |
|---|---|
| vehicle (n = 5) | 19.87951069747373 |
| BCAAs (n = 9) | 20.842711922133518 |
| mix 1 (n = 8) | 19.74378223588353 |
| mix 2 (n = 7) | 18.902677486741027 |
| mix 3 (n = 8) | 19.59261623197189 |Hind limb volume (mm3)
### Chart
| Category | |
|---|---|
| vehicle (n = 5) | 29.600599999999996 |
| BCAAs (n = 9) | 30.31975 |
| mix 1 (n = 8) | 32.7664 |
| mix 2 (n = 7) | 31.25375 |
| mix 3 (n = 8) | 31.526833333333332 |Hind limb vascularization (%)
C
Figure S1. Hind limb ultrasonography
(A) Sample ultrasound 3D images of the hind limb, reconstructed by VisualSonics 3D Software from multiple 2D frames previously acquired in Power Doppler mode. (B,C) Values of hind limb volume (mm3; (B)) and vascularization (%; (C)) measured at T4 in exercised mice treated with vehicle, BCAAs, mix 1, mix 2 or mix 3. Values are expressed as mean ± SEM from the number of mice indicated in brackets. No statistically significant differences between groups were found by one-way ANOVA followed by Bonferroni post hoc correction.

## Slide 3
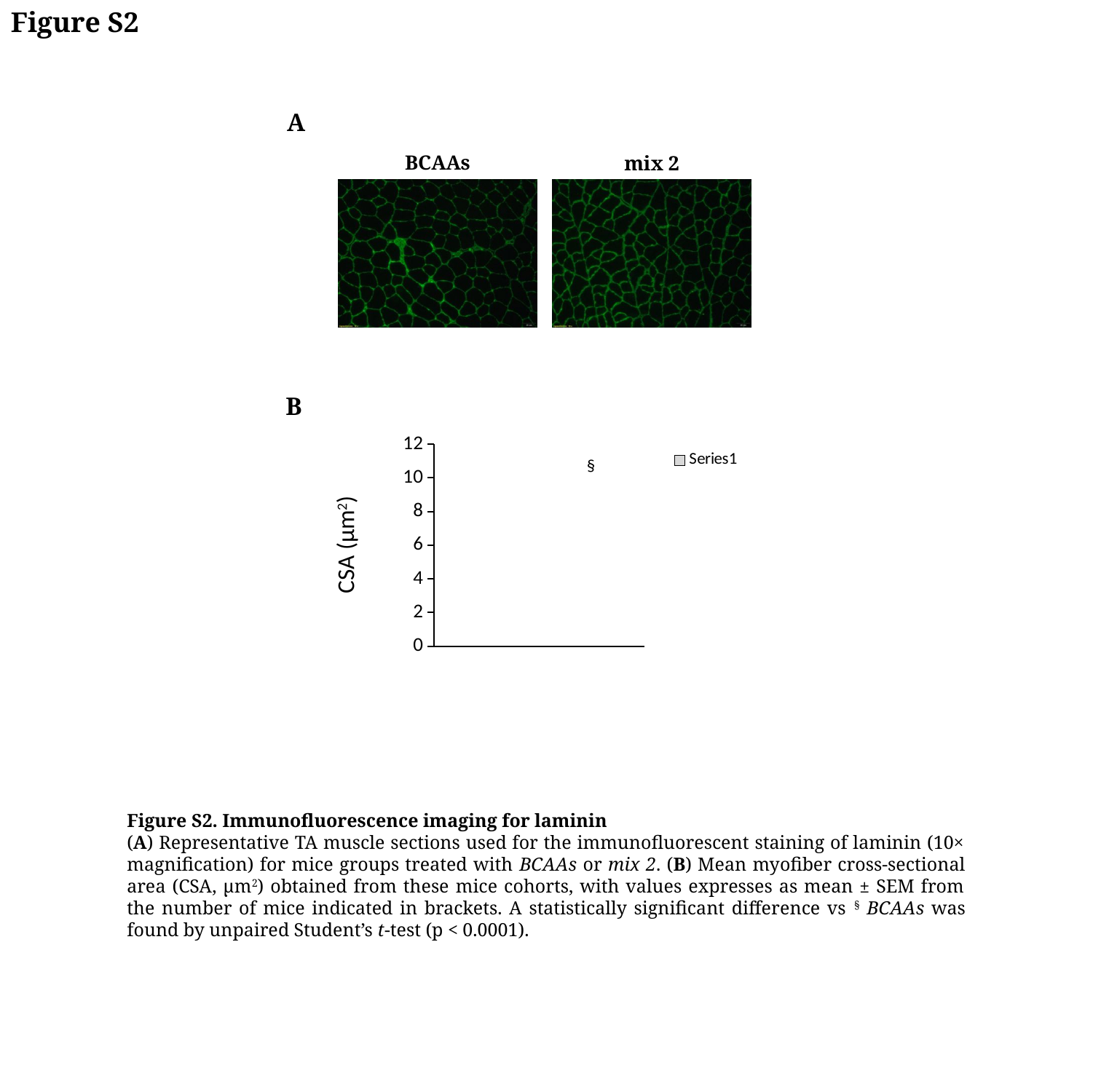

Figure S2
A
BCAAs
mix 2
B
### Chart
| Category | |
|---|---|
| BCAAs (n = 9) | 1942.0 |
| mix 2 (n = 7) | 2423.0 |CSA (µm2)
§
Figure S2. Immunofluorescence imaging for laminin
(A) Representative TA muscle sections used for the immunofluorescent staining of laminin (10× magnification) for mice groups treated with BCAAs or mix 2. (B) Mean myofiber cross-sectional area (CSA, µm2) obtained from these mice cohorts, with values expresses as mean ± SEM from the number of mice indicated in brackets. A statistically significant difference vs § BCAAs was found by unpaired Student’s t-test (p < 0.0001).

## Slide 4
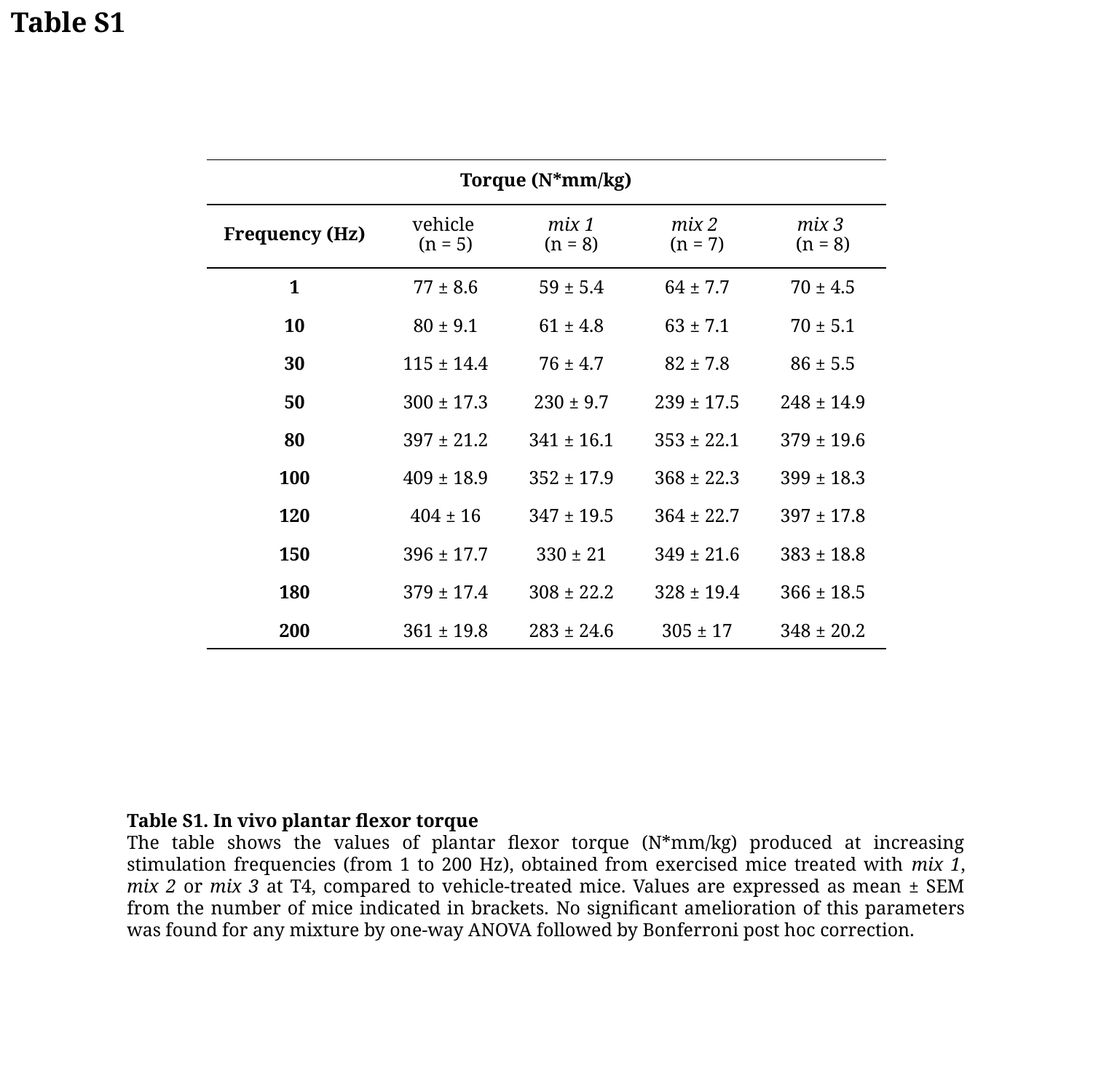

Table S1
| Torque (N\*mm/kg) | | | | |
| --- | --- | --- | --- | --- |
| Frequency (Hz) | vehicle (n = 5) | mix 1 (n = 8) | mix 2 (n = 7) | mix 3 (n = 8) |
| 1 | 77 ± 8.6 | 59 ± 5.4 | 64 ± 7.7 | 70 ± 4.5 |
| 10 | 80 ± 9.1 | 61 ± 4.8 | 63 ± 7.1 | 70 ± 5.1 |
| 30 | 115 ± 14.4 | 76 ± 4.7 | 82 ± 7.8 | 86 ± 5.5 |
| 50 | 300 ± 17.3 | 230 ± 9.7 | 239 ± 17.5 | 248 ± 14.9 |
| 80 | 397 ± 21.2 | 341 ± 16.1 | 353 ± 22.1 | 379 ± 19.6 |
| 100 | 409 ± 18.9 | 352 ± 17.9 | 368 ± 22.3 | 399 ± 18.3 |
| 120 | 404 ± 16 | 347 ± 19.5 | 364 ± 22.7 | 397 ± 17.8 |
| 150 | 396 ± 17.7 | 330 ± 21 | 349 ± 21.6 | 383 ± 18.8 |
| 180 | 379 ± 17.4 | 308 ± 22.2 | 328 ± 19.4 | 366 ± 18.5 |
| 200 | 361 ± 19.8 | 283 ± 24.6 | 305 ± 17 | 348 ± 20.2 |
Table S1. In vivo plantar flexor torque
The table shows the values of plantar flexor torque (N*mm/kg) produced at increasing stimulation frequencies (from 1 to 200 Hz), obtained from exercised mice treated with mix 1, mix 2 or mix 3 at T4, compared to vehicle-treated mice. Values are expressed as mean ± SEM from the number of mice indicated in brackets. No significant amelioration of this parameters was found for any mixture by one-way ANOVA followed by Bonferroni post hoc correction.

## Slide 5
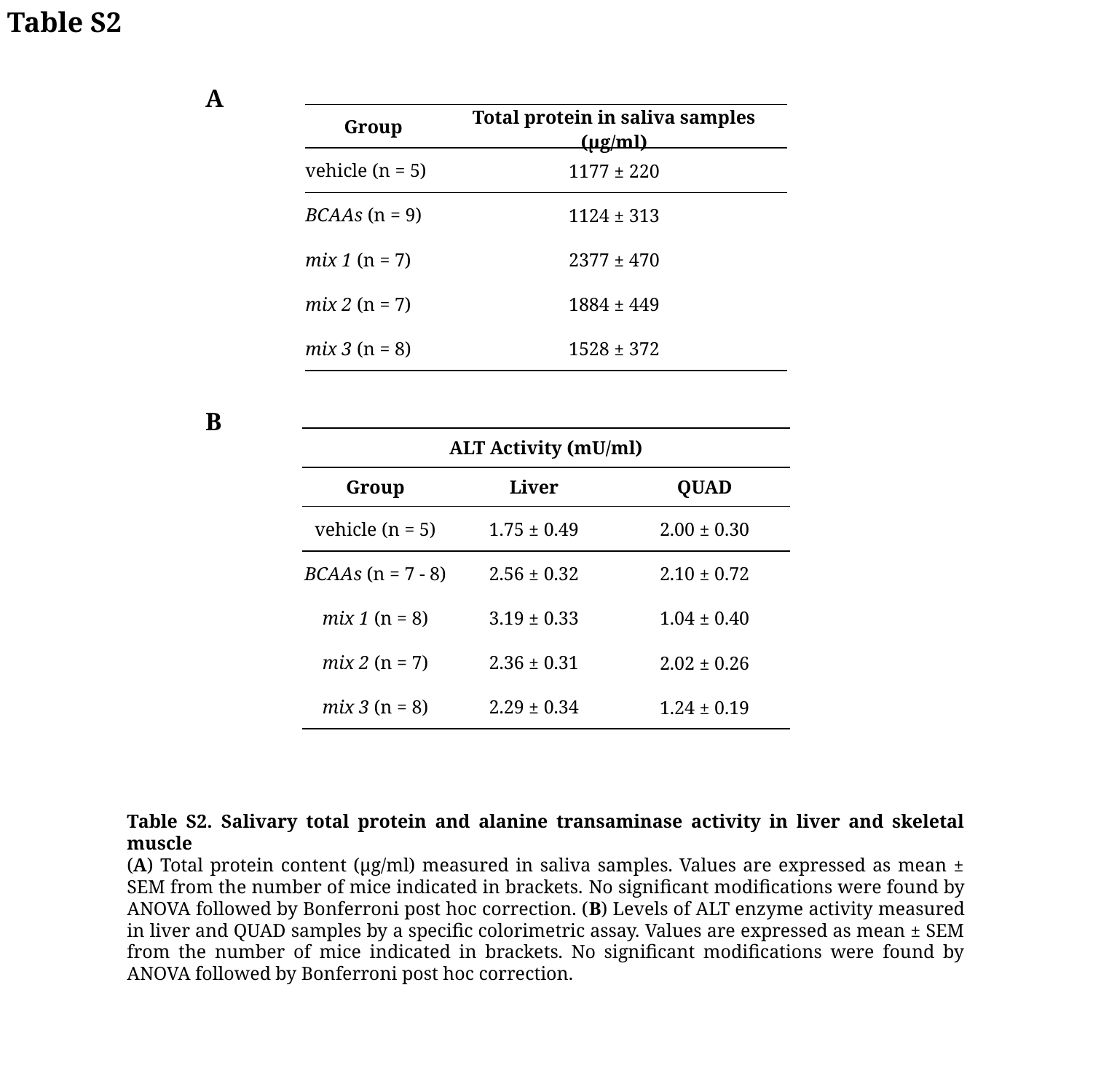

Table S2
A
| Group | Total protein in saliva samples (µg/ml) |
| --- | --- |
| vehicle (n = 5) | 1177 ± 220 |
| BCAAs (n = 9) | 1124 ± 313 |
| mix 1 (n = 7) | 2377 ± 470 |
| mix 2 (n = 7) | 1884 ± 449 |
| mix 3 (n = 8) | 1528 ± 372 |
B
| ALT Activity (mU/ml) | | |
| --- | --- | --- |
| Group | Liver | QUAD |
| vehicle (n = 5) | 1.75 ± 0.49 | 2.00 ± 0.30 |
| BCAAs (n = 7 - 8) | 2.56 ± 0.32 | 2.10 ± 0.72 |
| mix 1 (n = 8) | 3.19 ± 0.33 | 1.04 ± 0.40 |
| mix 2 (n = 7) | 2.36 ± 0.31 | 2.02 ± 0.26 |
| mix 3 (n = 8) | 2.29 ± 0.34 | 1.24 ± 0.19 |
Table S2. Salivary total protein and alanine transaminase activity in liver and skeletal muscle
(A) Total protein content (µg/ml) measured in saliva samples. Values are expressed as mean ± SEM from the number of mice indicated in brackets. No significant modifications were found by ANOVA followed by Bonferroni post hoc correction. (B) Levels of ALT enzyme activity measured in liver and QUAD samples by a specific colorimetric assay. Values are expressed as mean ± SEM from the number of mice indicated in brackets. No significant modifications were found by ANOVA followed by Bonferroni post hoc correction.
